# Heat-induced and spontaneous expression of *Hsp70.1Luciferase *transgene copies localized on Xp22 in female bovine cells

**DOI:** 10.1186/1756-0500-3-17

**Published:** 2010-01-22

**Authors:** Jean-Marc Lelièvre, Daniel Le Bourhis, Amandine Breton, Hélène Hayes, Jean-Luc Servely, Xavier Vignon

**Affiliations:** 1 INRA, UMR 1198 Biologie du Développement et Reproduction, F-78350 Jouy en Josas, France; 2 ENVA, UMR 1198 Biologie du Développement et Reproduction, F-78350 Jouy en Josas, France; 3 UNCEIA, Département R&D, 13, rue Jouet, F-94704 Maison-Alfort, France; 4 INRA, UMR 1313 Génétique Animale et Biologie Intégrative, F-78350 Jouy en Josas, France

## Abstract

**Background:**

Expression of several copies of the heat-inducible *Hsp70.1Luciferase *(*LUC*) transgene inserted at a single X chromosome locus of a bull (*Bos taurus*) was assessed in females after X-chromosome inactivation (XCI). Furthermore, impact of the chromosomal environment on the spontaneous expression of these transgene copies before XCI was studied during early development in embryos obtained after in vitro fertilization (IVF), when the locus was carried by the X chromosome inherited from the bull, and after somatic cell nuclear transfer (SCNT) cloning, when the locus could be carried by the inactive Xi or the active Xa chromosome in a female donor cell, or by the (active) X in a male donor cell.

**Findings:**

Transgene copies were mapped to bovine Xp22. In XX^*LUC *^female fibroblasts, i.e. after random XCI, the proportions of late-replicating inactive and early-replicating active X^*LUC *^chromosomes were not biased and the proportion of cells displaying an increase in the level of immunostained luciferase protein after heat-shock induction was similar to that in male fibroblasts. Spontaneous transgene expression occurred at the 8-16-cell stage both in transgenic (female) embryos obtained after IVF and in male and female embryos obtained after SCNT.

**Conclusions:**

The X^*LUC *^chromosome is normally inactivated but at least part of the inactivated X-linked *Hsp70.1Luciferase *transgene copies remains heat-inducible after random XCI in somatic cells. Before XCI, the profile of the transgenes' spontaneous expression is independent of the epigenetic origin of the X^*LUC *^chromosome since it is similar in IVF female, SCNT male and SCNT female embryos.

## Background

Menck and colleagues have reported a luminescent screening system based on the integration of a transgene composed of scaffold attachment regions flanking the murine *HSP70.1 *gene promoter linked to firefly luciferase cDNA [1]. Among the transgenic fetuses obtained, one male carried a cluster of 20 to 30 copies of the transgene [1]. Later, somatic cell nuclear transfer (SCNT) cloning with cells from this fetus generated a healthy and fertile bull for which we have localized the transgenic cluster on the X chromosome (this report). Thus an interesting animal model was available to investigate the inactivation/activation status of transgenes in bovine female fetuses from this bull. Indeed, dosage compensation between male and females is achieved after X-chromosome inactivation (XCI) in mammalian female cells, i.e. one of the two X, the inactive X (Xi) chromosome, is in great part transcriptionally silent [2,3]. At least in domestic mouse, XCI occurs in two waves early during development (reviewed in [2,3]). First, both X chromosomes are transcriptionally active during a short developmental window of the cleavage phase. Then, most studies agree that the paternally inherited X^P ^chromosome becomes inactivated by the blastocyst stage [2,3] and also in most placental cells. In the epiblast cells, both X chromosomes are again transiently active, before random XCI during gastrulation [4]. This results in a mosaic of two somatic cell types expressing X-linked genes inherited either from the mother or the father.

Some X-linked genes maintain bi-allelic expression in female cells [2,3]. On the human submetacentric X chromosome, 5% [5] to 15% [6] of the genes escape inactivation; they are preferentially found in clusters and more frequently on the short arm than on the long arm [6,7]. On the mouse acrocentric X chromosome, homologs of the human genes escaping inactivation are mostly inactivated and only two non-clustered genes with no homolog on the Y have been shown to escape inactivation [8]. Thus, the phenomenon of inactivation escape may depend on genomic context, including either the absence of sequence elements necessary for silencing spreading [9,10] or the presence of insulators/barriers that prevent XCI-coupled silencing [9,11].

Similarly, XCI-related silencing of X-linked transgenes may depend on the insertion site, the transgene's intrinsic properties or other unknown factors. Furthermore, it may vary between cell lineages or during development ([11-15] and references therein), as reported for 10% of the human X-linked genes [6,16] and one mouse X-linked gene [17].

Expression of an X-linked transgene has rarely been observed during early development and only in the domestic mouse [15,18] in which surprisingly, one X-linked transgene has been shown to display delayed expression when paternally inherited [15].

Analysis of SCNT cloned embryos can provide further insight on how XCI influences gene expression. For an Xi-associated transgene, silencing reversion has been reported in SCNT cloned early mouse embryos [18] but for an autosomal insertion, transgene-related and/or position effect-related silencing was found unchanged in SCNT cloned cattle [19].

Indirect evidence suggests that bovine and mouse XCI profiles are quite similar. De La Fuente and colleagues [20] have shown that in some cells from bovine embryos the two X chromosomes replicate asynchronously, one early and one late in S phase, thus XCI is established at the blastocyst stage 7 days after in vitro fertilization (IVF) in cattle. Indeed, late replicating regions including the Xi are generally transcriptionally inactive while early-replicating regions including the Xa are generally transcriptionally active (except in the mouse immediately after imprinted XCI; [3]). Furthermore, two reports clearly indicate that the paternally-inherited X is preferentially inactivated in the placenta [21] or in the chorion only [22], suggesting that imprinted XCI takes place earlier in the associated cell lineage(s), i.e., at least in the trophoblast, at the blastocyst stage. In somatic bovine cells, both X are inactivated, [21,22], suggesting that XCI occurs randomly in the bovine epiblast during gastrulation as in the mouse. To date, it has not been established whether some genes escape inactivation on the bovine X.

Several studies have shown that the expression of the *Hsp70.1Luciferase *transgene mimics that of the murine *Hsp70.1 *gene, *i.e*. the level of luciferase activity increases after heat-shock (HS) induction in both mouse and bovine transgenic embryonic and somatic cells and also spontaneously during embryonic genome activation (EGA) in early mouse transgenic embryos ([1] and references therein). To investigate whether some copies of the X-linked transgene remained inducible, we first analyzed the HS-induced luciferase activity and/or protein level in transgenic female somatic cells and blastocyst embryos. Second, we took advantage of the spontaneous activity of the transgene in early embryos before XCI and the relative success of SCNT cloning in cattle [23] to measure the influence on gene expression of the chromosomal environment inherited from spermatozoa or from male and female cells in early IVF embryos and SCNT embryos respectively.

## Materials and methods

All samples were generated according to the International Guiding Principles for Biomedical Research involving animals of experimental farms. The research work on cloned animals was approved by COMEPRA (Ethical and Precaution Committee for Agronomic Research Application) in December 1999.

The remaining of this section is found in [additional file 1: Material and methods].

## Results and discussion

### Transgenes are located on the X chromosome of the transgenic bull

**Figure 1:**
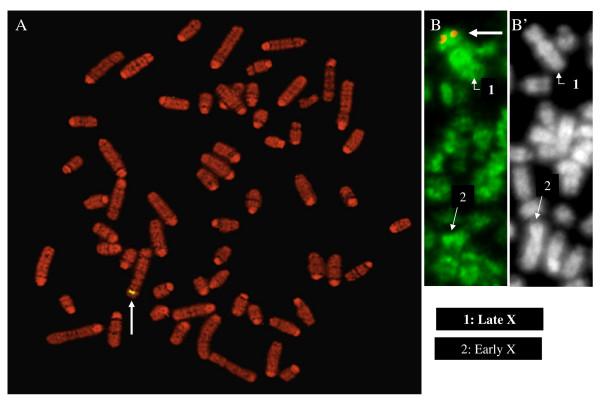
**The luciferase transgenes are located on bovine Xp22 and do not alter random X-chromosome inactivation**. (A) **A whole chromosome metaphase spread from the transgenic bull**. Late-replicating DNA of synchronized fibroblasts cells was labeled by BrdU. Metaphase spreads were prepared on slides, fixed with methanol/acetic acid (3:1) and then used for fluorescent in situ hybridization (FISH) using the biotin-labeled probe corresponding to the plasmid containing the whole 8 kb-long *Hsp70.1Luciferase *transgene. After DNA counterstaining with propidium iodide in alkaline conditions, BrdU-rich bands appear as dark chromosomal bands while early replicated R bands fluoresce red. In these conditions the transgenic locus was detected on the Xp22 band by immunolabeling of the biotin probe and immunodetection with FITC-conjugated secondary antibodies. No other signal was detected on complete metaphase spreads. (B) (B') **Partial metaphase spread of a female BSF731 fibroblast cell**. Metaphases were prepared as above before (B) immunodetection or (B') DNA counterstaining. (B) After DNA denaturation and FISH with the DIG-labeled transgene probe, late incorporation of BrdU was immunolabeled with anti-BrdU antibodies and immunodetected with FITC-conjugated secondary antibodies; the DNA probe was immunolabeled with anti-DIG antibodies and detected with TRITC-conjugated secondary antibodies; in the case shown, the transgene was localized on the late-replicating X chromosome. Arrows indicate the position of the transgene.

After hybridization with a probe specific for the whole transgene, a unique strong signal was observed on the short arm of the bovine submetacentric X in chromosome preparations from the transgenic bull, precisely in the early-replicating R band Xp22 (Figure 1A). The bovine Xp region is conserved with part of the human Xq [24,25] while the human Xp22 region, in which about 30% of the genes may escape inactivation [6], is conserved with part of the bovine Xq [24].

### In XX^LUC ^somatic female cells inactivation frequencies of both X are similar

Since XCI occurs randomly in the somatic bovine lineages [21,22], about half of the transgenic female somatic cells are expected to have an active X^*LUC *^chromosome inherited from the bull. Presence of a strong bias would indicate preferential inactivation of one of the X or preferential survival of the cells that inactivate one X. Although normal random XCI is reported for mouse and bovine clones [18,21,22,26], it was important to check whether this was the case in IVF females carrying the X of the transgenic bull since the bull was obtained by SCNT cloning, a technique which can result in developmental anomalies generally associated with abnormal epigenetic processes [23].

Analysis of metaphase chromosome spreads prepared from synchronized female XX^*LUC *^fibroblast cells cultured in the presence of BrdU during late S phase showed no bias. An equivalent number of BrdU-labeled X chromosomes or inactive Xi (N = 22) and partly BrdU-labeled X chromosomes or active Xa (N = 21) carried copies of the transgene. Furthermore, the transgenes' presence had no visible influence on Xp22 inactivation/activation since the X^*LUC*^p22 region replicates late on the Xi (Figure 1B) and on the normal X [27]. Thus, inactivation of the X carried by the bull's sperm is normal, which indicates that the presence of multiple copies of the transgene on either Xi or Xa is not toxic (counter-selected) to cell physiology and does not interfere with random XCI.

### Proportions of cells expressing the luciferase protein after heat-shock induction are similar in X^*LUC*^Y male and XX^*LUC *^female population of cultured fibroblasts

**Figure 2:**
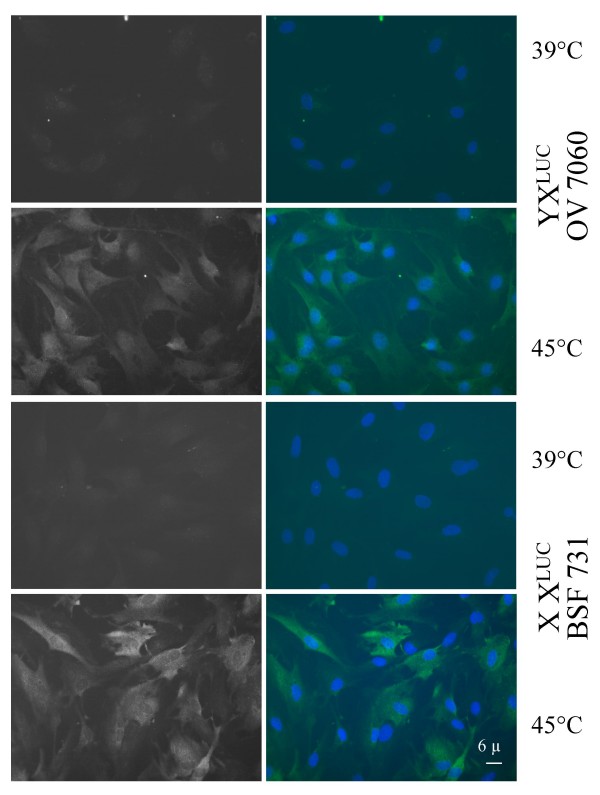
**Heat-induced immunostained luciferase proteins in bovine cells**. Male X^*LUC*^Y (OV7060) and female XX^*LUC *^(BSF731) transgenic fibroblast cells were fixed in cold methanol after continuous culture at 39°C or after 20 min at 45°C and 7 hours at 39°C. Immunostaining was performed with monoclonal mouse antibodies directed against the firefly luciferase and FITC-conjugated anti-mouse secondary antibodies (bright signal in left panels and green signal in right panels). Nuclear DNA was counterstained with Hoechst 3342 (blue signal on right panel). In these photographs (X400), 30 (97%) out of 33 OV7060 cells and 24 (82%) out of 29 BSF731 cells were counted positive after HS induction since they displayed a level of immunostaining two standard deviations above the mean value observed in untreated cells (see [additional File 2 Additional data]).

We compared the level of luciferase activity and protein after heat-shock (HS) induction in somatic female cells derived from two fetuses generated by IVF with the sperm of the transgenic original bull and referred to as F616 and BSF731 cells, and in male cells from the original transgenic bull (referred to as OV7060 cells). HS-induced luciferase activity ranged from 0.99 × 10^6 ^RLU.μg protein^-1^.min^-1 ^in female BSF731 fibroblast cells to about 2.60 × 10^6 ^RLU.μg protein^-1^.min^-1 ^in both male OV7060 and female F616 fibroblast cells (Table 1), corresponding to a many-fold increase in the three cell populations. Since in the BSF731 cells the numbers of Xi^*LUC *^and Xa^*LUC *^are similar after random XCI (see above), we compared the proportions of luciferase-positive cells after HS induction and immunostaining in this female cell population and the male OV7060 cell population (Table 1; Figure 2) They were very similar (Figure 2 and [additional File 2 Additional data]). It indicated that the X-linked luciferase transgene was expressed in most cells, strongly suggesting that at least some transgene copies remain active on the Xi in BSF731 cells.

**Table 1:** Heat-shock response vs. sex in bovine fibroblast cells carrying the *Hsp70.1Luciferase *transgenes

Origin of the fibroblast cultures	Sex	Mean luciferase specific activity^a ^RLU.μg protein^-1^.min^-1 ^± SD		Immunostained luciferase-positive cells after heat shock ^b^
			
		Control	After heat shock	
Transgenic adult bull "OV7060"	Male (X^*LUC*^Y)	205 ± 76	2.58 × 10^6 ^± 0.32 × 10^6^	92%

Transgenic fetus "BSF731"	Female (X^*LUC*^X)	315 ± 79	0.99 × 10^6 ^± 0.13 × 10^6^	80%

Transgenic fetus "F616"	Female (X^*LUC*^X)	875 ±160	2.62 × 10^6 ^± 0.35 × 10^6^	nd

^a ^RLU = relative light unit.

^b ^percentage of cells, which after HS induction displayed cytoplasmic immunostaining values two standard deviations above the mean value observed in non-induced cells.

In tissue biopsies of three fetal organs tested from the F616 female fetus, a strong increase in the level of luciferase activity after HS-induction was observed (Table 2). Overall, these data strongly suggest that the presence of 20 to 30 transgene copies [1] does not prevent the HS-induced expression of at least some of them in the different cells and tissues tested, whether they are on the Xi or Xa.

**Table 2:** Rate of increase in luciferase activity in fetal and placental female tissues after heat shock^a^

Origin of biopsy^b^	Rate of increase after HS^c^
Placenta	38

Heart	141

Muscle	282

Lung	2930

^a ^the level of specific luciferase activity (RLU.μg protein^-1^.min^-1^) was measured in explants of the transgenic female F616 fetus after HS induction and compared with the level measured in non-induced explants.

^b ^all biopsies were recovered from the transgenic female F616 fetus.

^c ^average value based on the measurement of luciferase activity on two independent extracts for each tissue and each condition.

### The pattern of spontaneous *Hsp70.1Luciferase *transgene activity during early development was conserved in all bovine embryos

**Figure 3:**
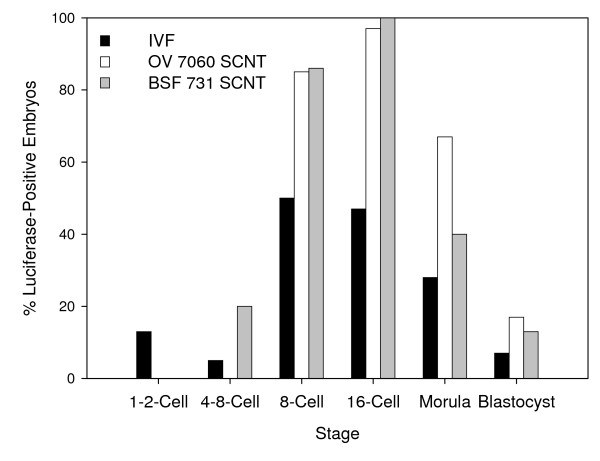
**Evolution of the percentage of luciferase-positive embryos in early IVF and SCNT transgenic bovine embryos**. In vitro matured bovine oocytes were used to obtain IVF embryos (black bars) after in vitro fertilization with the semen of the transgenic OV7060 X^*LUC*^Y bull, and male X^*LUC*^Y OV7060 (open bars) and female X^*LUC*^X (grey bars) SCNT embryos after nuclear transfer of fibroblast cells. Embryos were harvested individually at the correct stage and their spontaneous (in the absence of heat-shock induction) luciferase activity measured. Embryos were considered luciferase-positive when the level of luciferase activity was above the background level. The percentage of positive embryos increased at the 8-16-cell stage in all embryo types.

During normal development, between the one-cell and the 4-8-cell stages, the level of luciferase activity was null in at least 80% of the IVF and SCNT transgenic bovine embryos and very low in the remaining 20% (Figure 3; Table 3). Since the sperm used here was obtained from a bull hemizygous at the X-linked transgenes, statistically half of the IVF embryos, all female, should be transgenic. As expected, about 50% of the "8-16-cell" IVF embryos displayed a high level of luciferase activity at days 3 and 4, suggesting that the transgenes are expressed in most female IVF embryos. At the morula stage, 32% of the IVF embryos remained luciferase-positive but the level of luciferase activity per embryo had already decreased significantly (P < 0.05). At the blastocyst stage, less than 10% of the IVF embryos displayed luciferase activity, and all at a low level. Moreover, the percentages of luciferase-positive embryos differed significantly between the 8-16-cell stage and the 4-8-cell or blastocyst stages in the three embryo types (Table 3).

**Table 3:** Changes in luciferase activity and in the percentage of luciferase-positive embryos during early development

Embryo type		Stage					
		
		1-2-cell	4-8-cell	8-cell	16-cell	Morula	Blasto.
Female IVF	Number of embryos	36	20	24	30	32	28
	
	Luciferase activity (RLU. min^-1^. embryo^-1^)	325 ± 175*	70	20940 ± 5790	36540 ± 8900	3000 ± 1880	370 ± 330
	
	% positive embryos	11%^a^	5% ^a^	50% ^b^	47% ^b^	28% ^ab^	7% ^a^

Male SCNT	Number of embryos	-	13	27	33	9	12
	
	Luciferase activity (RLU. min^-1^. embryo^-1^)	-	0	33500 ± 7056	13700 ± 3120	1400 ± 446	480 ± 165
	
	% positive embryos	-	0% ^a^	85% ^b^	97% ^b^	67% ^c^	17% ^a^

Female SCNT	Number of embryos	-	20	21	39	10	8
	
	Luciferase activity (RLU. min^-1^. embryo^-1^)	-	515 ± 140	2240 ± 300	9915 ± 1490	525 ± 337	245
	
	% positive embryos	-	16% ^a^	86% ^b^	100% ^b^	40% ^a^	13% ^a^

^abc ^different letters indicate that values on the line are significantly different (P < 0.05).

* SEM; SEM could not be calculated when 0 or only one embryo displayed a level of luciferase activity above the background level.

Before nuclear transfer, the BSF731 female donor cells carried the transgenes either on the Xa or the Xi with a similar probability (see above). However, in female BSF731-derived SCNT embryos, we found no evidence for two sub-populations displaying two different levels and patterns of luciferase activity and the standard error to the mean was similar to that observed in the two other embryo types (Table 3). This indicates that after cloning, the number of active genes was similar in these female SCNT embryos as expected if their XCI-dependent silencing in the donor cell was not achieved (as suggested from Table 1; Figure 2) or was reverted after cloning [18]. In turn, the variation in HS response of BSF731 fibroblast cells may result either from a variable number of inactivated transgenes on the Xi or from a variable HS response in the BSF731 cell population.

Since the *Hsp70.1Luciferase *transgene can be similarly active at the 8-16-cell stage in both IVF and cloned embryos, this further suggests that neither the paternal origin of the transgenic X^*LUC *^in the case of IVF embryos, nor the origin, male or female, of the somatic cells, in the case of SCNT embryos, prevented spontaneous, oocyte-driven, expression of the X-linked transgenes.

We detected an increased level of luciferase activity after heat-shock induction in IVF female blastocysts [additional File 3 Additional Table S1] and placental tissue (Table 2) in which imprinted XCI, i.e. inactivation of the paternally-inherited X^*LUC*^, is expected. However, we cannot yet conclude whether the transgenes escaped inactivation after imprinted XCI since the presence of cells in which XCI had not occurred or had occurred randomly is likely.

In conclusion, using a species other than mouse and different approaches we have investigated the expression of an X-linked transgene to determine its innocuousness as well as that of the transgene insertion site, and to test its sensitivity to XCI-dependent or -independent silencing. The results indicate that the transgenic X inherited from the cloned bull is normally inactivated/activated in somatic female cells and that at least some of the transgene copies at this locus escape XCI-coupled silencing in these cells. Whether this is due to HS-dependent [28,29] or HS-independent [30] properties of the transgene, to the insertion site and/or to the creation of a new genomic environment remains to be determined.

## Competing interests

The authors declare that they have no competing interests.

## Authors' contributions

DLB and XV generated the bovine bull; XV and JLS generated cell cultures, JML, AB, MC, HH and JLS generated IVF embryos, performed FISH and immuno-detection, and measured luciferase activity and protein concentrations; DLB generated SCNT embryos, JML designed the experiments, performed statistical tests and wrote the draft. HH and XV edited the draft and all authors agreed on the final version of this report.

## Supplementary Material

Additional file 1 Material and methods Click here for file

Additional file 2 Additional data Click here for file

Additional file 3 Additional Table S1 Click here for file

## References

[B1] Menck M Mercier Y Campion E Lobo RB Heyman Y Renard JP Thompson EM Prediction of transgene integration by noninvasive bioluminescent screening of microinjected bovine embryos Transgenic Res 1998 7 331 341 10.1023/A:1008841222138 9859222

[B2] Payer B Lee JT X chromosome dosage compensation: how mammals keep the balance Annu Rev Genet 2008 42 733 772 10.1146/annurev.genet.42.110807.091711 18729722

[B3] Heard E Disteche CM Dosage compensation in mammals: fine-tuning the expression of the X chromosome Genes Dev 2006 20 1848 1867 10.1101/gad.1422906 16847345

[B4] Mak W Nesterova TB de Napoles M Appanah R Yamanaka S Otte AP Brockdorff N Reactivation of the paternal X chromosome in early mouse embryos Science 2004 303 666 669 10.1126/science.1092674 14752160

[B5] Johnston CM Lovell FL Leongamornlert DA Stranger BE Dermitzakis ET Ross MT Large-scale population study of human cell lines indicates that dosage compensation is virtually complete PLoS Genet 2008 4 e9 10.1371/journal.pgen.0040009 18208332 PMC2213701

[B6] Carrel L Willard HF X-inactivation profile reveals extensive variability in X-linked gene expression in females Nature 2005 434 400 404 10.1038/nature03479 15772666

[B7] Tsuchiya KD Willard HF Chromosomal domains and escape from X inactivation: comparative X inactivation analysis in mouse and human Mamm Genome 2000 11 849 854 10.1007/s003350010175 11003698

[B8] Chow JC Yen Z Ziesche SM Brown CJ Silencing of the mammalian X chromosome Annu Rev Genomics Hum Genet 2005 6 69 92 10.1146/annurev.genom.6.080604.162350 16124854

[B9] Tsuchiya M Piras V Choi S Akira S Tomita M Giuliani A Selvarajoo K Emergent genome-wide control in wildtype and genetically mutated lipopolysaccarides-stimulated macrophages PLoS One 2009 4 e4905 10.1371/journal.pone.0004905 19300509 PMC2654147

[B10] Carrel L Park C Tyekucheva S Dunn J Chiaromonte F Makova KD Genomic environment predicts expression patterns on the human inactive X chromosome PLoS Genet 2006 2 e151 10.1371/journal.pgen.0020151 17009873 PMC1584270

[B11] Li N Carrel L Escape from X chromosome inactivation is an intrinsic property of the Jarid1c locus Proc Natl Acad Sci USA 2008 105 17055 17060 10.1073/pnas.0807765105 18971342 PMC2579377

[B12] Hadjantonakis AK Cox LL Tam PP Nagy A An X-linked GFP transgene reveals unexpected paternal X-chromosome activity in trophoblastic giant cells of the mouse placenta Genesis 2001 29 133 140 10.1002/gene.1016 11252054

[B13] Ciavatta D Kalantry S Magnuson T Smithies O A DNA insulator prevents repression of a targeted X-linked transgene but not its random or imprinted X inactivation Proc Natl Acad Sci USA 2006 103 9958 9963 10.1073/pnas.0603754103 16777957 PMC1479543

[B14] Goldman MA Reeves PS Wirth CM Zupko WJ Wong MA Edelhoff S Disteche CM Comparative methylation analysis of murine transgenes that undergo or escape X-chromosome inactivation Chromosome Res 1998 6 397 404 10.1023/A:1009229423535 9872669

[B15] Tam PP Williams EA Tan SS Expression of an X-linked HMG-lacZ transgene in mouse embryos: implication of chromosomal imprinting and lineage-specific X-chromosome activity Dev Genet 1994 15 491 503 10.1002/dvg.1020150608 7834909

[B16] Carrel L Willard HF Heterogeneous gene expression from the inactive X chromosome: an X-linked gene that escapes X inactivation in some human cell lines but is inactivated in others Proc Natl Acad Sci USA 1999 96 7364 7369 10.1073/pnas.96.13.7364 10377420 PMC22091

[B17] Sheardown S Norris D Fisher A Brockdorff N The mouse Smcx gene exhibits developmental and tissue specific variation in degree of escape from X inactivation Hum Mol Genet 1996 5 1355 1360 10.1093/hmg/5.9.1355 8872477

[B18] Eggan K Akutsu H Hochedlinger K Rideout W Yanagimachi R Jaenisch R X-Chromosome inactivation in cloned mouse embryos Science 2000 290 1578 1581 10.1126/science.290.5496.1578 11090356

[B19] Bordignon V Keyston R Lazaris A Bilodeau AS Pontes JH Arnold D Fecteau G Keefer C Smith LC Transgene expression of green fluorescent protein and germ line transmission in cloned calves derived from in vitro-transfected somatic cells Biol Reprod 2003 68 2013 2023 10.1095/biolreprod.102.010066 12606490

[B20] De La Fuente R Hahnel A Basrur PK King WA X inactive-specific transcript (Xist) expression and X chromosome inactivation in the preattachment bovine embryo Biol Reprod 1999 60 769 775 10.1095/biolreprod60.3.769 10026129

[B21] Xue F Tian XC Du F Kubota C Taneja M Dinnyes A Dai Y Levine H Pereira LV Yang X Aberrant patterns of X chromosome inactivation in bovine clones Nat Genet 2002 31 216 220 10.1038/ng900 12032569

[B22] Dindot SV Farin PW Farin CE Romano J Walker S Long C Piedrahita JA Epigenetic and Genomic Imprinting Analysis in Nuclear Transfer Derived Bos gaurus/Bos taurus Hybrid Fetuses Biol Reprod 2004 71 470 478 10.1095/biolreprod.103.025775 15044262

[B23] Yang X Smith SL Tian XC Lewin HA Renard JP Wakayama T Nuclear reprogramming of cloned embryos and its implications for therapeutic cloning Nat Genet 2007 39 295 302 10.1038/ng1973 17325680

[B24] Rubes J Kubickova S Musilova P Amaral ME Brunner RM Goldammer T Assignment of chromosome rearrangements between X chromosomes of human and cattle by laser microdissection and Zoo-FISH Chromosome Res 2005 13 569 574 10.1007/s10577-005-0982-9 16170621

[B25] Goldammer T Amaral ME Brunner RM Owens E Kata SR Schwerin M Womack JE Clarifications on breakpoints in HSAX and BTAX by comparative mapping of F9, HPRT, and XIST in cattle Cytogenet Genome Res 2003 101 39 42 10.1159/000073416 14571135

[B26] Nolen LD Gao S Han Z Mann MR Gie Chung Y Otte AP Bartolomei MS Latham KE X chromosome reactivation and regulation in cloned embryos Dev Biol 2005 279 525 540 10.1016/j.ydbio.2005.01.016 15733677

[B27] Coppola G Pinton A Joudrey EM Basrur PK King WA Spatial distribution of histone isoforms on the bovine active and inactive X chromosomes Sex Dev 2008 2 12 23 10.1159/000117715 18418031

[B28] Brown SA Kingston RE Disruption of downstream chromatin directed by a transcriptional activator Genes Dev 1997 11 3116 3121 10.1101/gad.11.23.3116 9389644 PMC316745

[B29] Inouye S Fujimoto M Nakamura T Takaki E Hayashida N Hai T Nakai A Heat Shock Transcription Factor 1 Opens Chromatin Structure of Interleukin-6 Promoter to Facilitate Binding of an Activator or a Repressor Journal of Biological Chemistry 2007 282 33210 33217 10.1074/jbc.M704471200 17766920

[B30] Thompson EM Christians E Stinnakre MG Renard JP Scaffold attachment regions stimulate HSP70.1 expression in mouse preimplantation embryos but not in differentiated tissues Mol Cell Biol 1994 14 4694 4703 8007971 10.1128/mcb.14.7.4694-4703.1994 PMC358842

