# Supplementary material for: Heat-induced and spontaneous expression of Hsp70.1Luciferase transgene copies localized on Xp22 in female bovine cells
Source: BMC Res Notes. 2010 Jan 22;3:17. doi: 10.1186/1756-0500-3-17 (PMC2832894; doi:10.1186/1756-0500-3-17)
Supplement: Additional file 1 — Material and methods [file 1756-0500-3-17-S1.DOC]

# Additional files

## Additional file 1

## File format: DOC

## Title: Lelievre_Additional _File1

Description: Material and methods

# Material and Methods

All samples were generated according to the International Guiding Principles for Biomedical Research involving animals of experimental farms. The research work on cloned animals was approved by the INRA Ethics Committee (COMEPRA, Ethical and Precaution Committee for Agronomic Research Application) in December 1999.

##### In vitro maturation of oocytes

All the procedures have been described previously [1]. When necessary, matured metaphase II oocytes were separated in two batches, one for in vitro fertilization (IVF) and one for somatic cell nuclear transfer (SCNT).

## In vitro fertilization, embryo culture and transfer of transgenic embryos

*In vitro* fertilization, embryo cultures and transfer of transgenic embryos were performed as described elsewhere [1] using semen from the SCNT transgenic bull. Briefly, after in vitro maturation metaphase-II oocytes were co-cultured with heparin-capacitated thawed spermatozoa of the transgenic bull during 18-22 h. Subsequently, after removal of cumulus cells by vortexing, fertilized oocytes were co-cultured for 7 days at 39°C in a humidified chamber with 5% CO2 and air in micro-droplets of B2 culture medium with a feeder layer of monkey liver Vero cells [1]. Luciferase positive embryos were identified by measuring as described below the luciferase activity of biopsies after heat shock at the morula stage. Day-7 embryos were transferred into synchronized recipients cows and two 70 days-old fetuses, named F616 and BSF731 were recovered.

# Origin, culture and preparation of fibroblast cells for cloning and immunostaining

F616 and BSF731 female fibroblast cultures were initiated from skin//muscle explants of 70 days-old fetuses obtained as described above. OV7060 male bovine fibroblast cultures were derived from the transgenic bull’s ear biopsies. One to two mm sliced explants were aseptically recovered and cultured in plastic Petri dishes in Dulbecco's Modified Eagle Medium (DMEM) supplemented with 10% fetal calf serum (FCS) and antibiotics at 39°C in a humidified chamber with 5% CO2 and air. Confluent cells were recovered after trypsin treatment, which defines a ’passage’, and subsequently cultured in a new plate after a 1/3 dilution in fresh culture medium, or frozen after resuspension in concentrated FCS. Cells used for cloning corresponded to frozen stocks of passage 3 (BSF731G) and passage 5 (OV7060). For immunofluorescence, cells were plated on SuperFrost slides. For SCNT, confluent fibroblasts were kept for another four days before nuclear transfer in fresh DMEM medium supplemented with 10% FCS.

## SCNT protocol

Our standard nuclear transfer protocol [2] was used to generate all - except the OV7060 bull (see below) - SCNT embryos described in this study. This protocol is based on the simultaneous fusion of the donor fibroblast cell in G0 phase with a non-activated enucleated oocyte and activation of the reconstructed embryo; immediately after electrofusion, reconstituted embryos were activated in the presence of 5 µM cytochalasin and 10 µg/ml cycloheximide. After 5h of activation, embryos were rinsed and then cultured in B2 medium as described above.

**SCNT protocol initially used to generate the transgenic bull**

Transgenic bovine fibroblast cells derived from the transgenic F11 fetus have been described elsewhere [1] and were used as donor cells for fusion with enucleated mature oocytes that were pre-activated as described previously [3]. Reconstituted embryos were co-cultivated in micro-droplets of B2 culture medium on monkey liver Vero cells [1] as feeding layers for in vitro development of embryos under mineral oil. After 7 days of culture, blastocyst embryos were transferred into recipients cows synchronized by hormonal treatments [1]. One healthy bull was obtained [3].

## Heat shock treatments, measurement of luciferase activity and immunostaining of the luciferase protein

Intercotyledonary placenta and somatic fragments were removed from F616 fetus, respectively. Heart, leg, lung and intercotyledonary explants of 20-40 mg were aseptically recovered and transferred to 1 ml of M199 Hepes medium and cultured in a humidified chamber with 5% CO2 and air at 39°C continuously or after heat shock. Both types of explants were further cultured for 6 hours at 39°C and finally frozen in liquid nitrogen. Fragments were directly grounded in microtubes with a rotating pestle and the powder resuspended in 250-500 µl of lysis buffer [4]. For each temperature condition, the measurement of luciferase activity was performed on two independent extracts (replicates) for each tissue of the F616 fetus and placenta. IVF embryos were harvested at 20 (1 cell), 28 (2 cells), 46 (4-8 cell), 72 (about 8 cells), 92 (about 16 cells) hours, and 5 (morula) and 7 days (blastocyst) post-insemination. SCNT embryos underwent cleavage at 20-24 hours post-fusion. They were harvested at 40 (4-8-cell), 72 (8-cell), 92 (16-cell) hours, and 5 (morula) and 7 (blastocyst) days post-fusion. Embryos were all frozen individually in a microtube without extraction buffer. Fifty µl of extraction buffer [4] were immediately added during thawing. Luciferase activity was measured using a luminometer as described earlier [1]. Because the sperm used during this study was obtained from a hemizygous bull for the transgenic locus, half of the IVF embryos were statistically transgenic. At the 8 to 16-cell stage or after heat shock, we indeed found that on average about 50% of embryos displayed luciferase activity. Therefore the remaining embryos were taken to be non-transgenic and were not considered at this stage. At other stages, the mean levels of luciferase were also calculated by only taking account of luciferase values above the background level. Heat shock (HS) was performed in a water bath at 45°C for 15 min at the blastocyst stage and 25 min for tissue explants.

Fibroblasts were cultured in plastic wells at an initial density corresponding to 33% confluence. Heat shock was performed at 45°C in a water bath for 20 min and cells were then returned to 39°C for 6 to 7 hours. This was sufficient for both luciferase measurements and immunocytological detection. Cells were rinsed in PBS and then scrapped with 0.5 ml of lysis buffer. After 1 min, the extracts were frozen. For immunocytology, cells were cultured on coverslips in the same conditions, fixed with cold methanol for 10 min, rinsed in PBS and directly blocked in PBS / 1% bovine serum albumin and 0.05% Tween® 20 before immunodetection. We used a 75-fold dilution of monoclonal mouse antibodies directed against firefly luciferase (Sigma) and a 400-fold dilution of FITC-conjugated antibodies directed against the IgG fraction of mouse antibodies (Sigma). After DNA counterstaining with Hoechst 33342, coverslips were drained and mounted on slides with Vectashield®. Fibroblast cells were observed using digital imaging microscopy with an epifluorescence microscope (Zeiss Axiovert) and x40 oil immersion objectives. Fibroblast images were recorded digitally with a high resolution CDD camera using IPLab imaging software (BD, Franklin Lakes, NJ USA). Grayscale images were pseudo-colored after capture. To determine the number of responsive, luciferase-positive transgenic cells, the images of non-treated immunostained male or female cells (about 30 cells per view) were recorded in conditions to minimize the signal due to immunostaining; next, these conditions were used to record the images of heat-shocked cells. Positive cells, i.e. cells immunostained by anti-luciferase proteins were identified visually and using the histogram plugin of the ImageJ software [5]. The average level of signal (on the scale ranging from 0 to 255 arbitrary units) of an area of 700 to 1000 pixels was determined in the most intensely immunostained region of the cytoplasm of each cell. In these conditions, with or without heat shock, non-transgenic bovine fibroblast cells displayed a similar level of background immunostaining signals below 70. The maximum level of immunostaining signal in transgenic BSF731 cells varies from 54 to 160 after HS induction and from 55 to 70 without HS. In the example shown (Fig.2 and see [additional File 2 Additional Data]), its maximum is 70±3 and the average level in the cell population is 66±3 in non-treated transgenic cells; in treated cells, the maximum level is 153±17. If cells are considered positive when the immunostaining level is two standard deviations above the mean level, 82% cells are positive. Three to four hundreds transgenic cells obtained in three different experiments were analyzed in each case

## Protein dosage in cell extracts

The amount of proteins was determined in extracts from fibroblast cells and tissue explants according to the bicinchoninic acid assay and the recommendations of the manufacturer (Uptima Interchim).

## FISH analysis and BrdU detection

Metaphase chromosomes for R-banding were prepared, hybridized and observed essentially as described elsewhere [6]. Briefly, fibroblast cells were synchronized in S phase by thymidine excess in the culture medium for 14-16 hours. After thymidine removal, cells were cultured in the presence of 100 µM BrdU, leading to BrdU incorporation in chromosomal regions replicated during the second half of the S phase [6]. Probes were prepared from the plasmid used for creating the transgenic bull [1] by nick translation using commercial kits. Biotin-dATP or Digoxigenin DIG-dUTP was used for labeling.

To visualize more easily the active early-replicating and the inactive late-replicating X chromosomes, a 4 min denaturation step was performed at 74°C in 50% formamide, followed by hybridization using the standard protocol. The FISH and BrdU signals were detected using sheep IgG antibodies directed against DIG (Roche) and TRITC-coupled antibodies directed against the sheep IgG fraction, and mouse antibodies directed against BrdU (Becton-Dickinson) and FITC-coupled antibodies directed against the mouse IgG fraction (Sigma) respectively. DNA was counterstained with Hoechst 33342. After mounting, slides were observed with a standard epifluorescence Zeiss microscope equipped with a 50 W UV lamp and a digital camera controlled by the IPLab software (BD Biosciences Bioimaging, Rockville, USA).

## Statistical analysis

Since the highest background level was estimated at 1200 RLU.mn^-1^, this value was subtracted from the measured values of luciferase activity. The mean levels of luciferase activity were calculated by only taking account of luciferase values above the background level. Quantitative results were analyzed using one-way ANOVA with the SYSTAT software and Bonferoni test (SYSTAT Software GmbH, Erkrath, Germany). The χ^2^ test was used to compare embryo populations.

# References

1. Menck M, Mercier Y, Campion E, Lobo RB, Heyman Y, Renard JP, Thompson EM: **Prediction of transgene integration by noninvasive bioluminescent screening of microinjected bovine embryos.** *Transgenic Res* 1998, **7:**331-341.

2. Gall L, Le Bourhis D, Ruffini S, Boulesteix C, Vignon X: **Unexpected nuclear localization of Cdc25C in bovine oocytes, early embryos, and nuclear-transferred embryos.** *Reproduction* 2008, **135:**431-438.

3. Vignon X, Chesne P, Le Bourhis D, Flechon JE, Heyman Y, Renard JP: **Developmental potential of bovine embryos reconstructed from enucleated matured oocytes fused with cultured somatic cells.** *C R Acad Sci III* 1998, **321:**735-745.

4. Thompson EM, Christians E, Stinnakre MG, Renard JP: **Scaffold attachment regions stimulate HSP70.1 expression in mouse preimplantation embryos but not in differentiated tissues.** *Mol Cell Biol* 1994, **14:**4694-4703.

5. **ImageJ** [ http://rsb.info.nih.gov/ij/]

6. Popescu CP, Hayes H, Dutrillaux B: *Techniques de cytogénétique animale.* Paris: INRA Editions; 1998.
