# Supplementary material for: Heat-induced and spontaneous expression of Hsp70.1Luciferase transgene copies localized on Xp22 in female bovine cells
Source: BMC Res Notes. 2010 Jan 22;3:17. doi: 10.1186/1756-0500-3-17 (PMC2832894; doi:10.1186/1756-0500-3-17)
Supplement: Additional file 3 — Additional Table S1 [file 1756-0500-3-17-S3.DOC]

## Additional file 3

## File format: DOC

## Title: Lelievre_Additional _File3

Description: Additional Table S1

## Table S1 - Heat-induced luciferase activity in transgenic female IVF and SCNT embryos at the blastocyst stage

| Origin of embryos | Without heat shock | | | After heat shock | | |
| --- | --- | --- | --- | --- | --- | --- |
|  | Number of embryos | Mean Luciferase  Activity  RLU.mn^-1^.embryo^-1^  ± SEM | % Luciferase-positive Embryos  (N embryos) | Number of embryos | Mean Luciferase  Activity  RLU.mn^-1^.embryo^-1^  ± SEM | % Luciferase-positive embryos  (N embryos) |
| IVF^b^ | 30 | 700^a^ | 3%  (n =1) | 40 | 64070  ± 8020 | 45%  (n= 18) |
| Female SCNT^b^ | 10 | 245 ^a^ | 10%  (n=1) | 4 | 7580  ±765 | 100%  (n=4) |

^a^ SEM could not be calculated since only one embryo displayed a level of luciferase activity above the background level

^b^ IVF embryos were obtained after in vitro fertilization with the transgenic bull’s sperm while SCNT embryos were derived from BSF731 transgenic female cells.
